# Mutagenesis in the Age of Next-Generation-Sequencing and Genome Editing

**DOI:** 10.3390/plants12193403

**Published:** 2023-09-27

**Authors:** Zhanguo Xin

**Affiliations:** Plant Stress and Germplasm Development Unit, USDA-ARS, 3810 4th Street, Lubbock, TX 79415, USA; zhanguo.xin@ars.usda.gov; Tel.: +01-806-749-5560

Mutagenesis is a proven, classical technique for inducing a broad spectrum of DNA variations and has led to the creation of thousands of improved varieties in many crop species [1]. With the advent of molecular biology, mutagenesis has played a key role in the elucidation of the mechanisms of plant adaptation, growth and development, metabolic pathways, and signal transduction. Mutagenesis, especially when it is chemically induced, can introduce thousands of random mutations in a genome and create many scorable phenotypes. With the ever increasing sequencing data output and quality along with its decreased price, it is now possible to sequence a sizable collection of induced mutants to reveal mutations in most genes in a genome [2]. The sequenced mutant library not only serves as an important resource for validating gene functions through reverse genetics, but can also serve as efficient resource to identify valuable gene mutations for crop improvement by cross-referencing beneficial gene mutations identified in other crop species. A high-quality mutant library often displays a wide diversity of mutant phenotypes [3,4], providing a rich resource for the identification of genes underlying various phenotypes through a forward genetics approach. This is traditionally carried out through map-based cloning, which can be a lengthy and expensive process [5]. Advancements in next-generation sequencing have enabled the simultaneous generation and mapping of millions of DNA markers to identify the causal mutations for the phenotype (trait) of interest at an affordable price [6,7,8]. With reverse and forward genetics, a high-quality mutant library can contain many novel traits for breeding and provide targets for efficient genome editing. 

A critical drawback of an induced mutant library is the high density of mutations unrelated to the phenotype of interest, i.e., the causal mutation [3,9]. The mutations unlinked to the causal mutation can be removed using recurrent backcrosses; however, it often requires several rounds of backcrosses, and the mutations that are linked to the causal mutation can be difficult to remove. The presence of a high amount of background mutations often prevents the direct use of mutants in plant breeding programs. Fortunately, the rapid and exciting progress in genome editing in the last decade promises to revolutionize plant breeding [10]. However, it is still unfeasible to create multitudes of mutations in each gene in a genome to determine if the mutations are beneficial to agronomically important traits, such as yield. Genome editing can be more efficient if the targeted mutations are known. With the continuous improvement in sequencing technologies and the decrease in sequencing costs, it has already become feasible to rapidly screen useful traits and efficiently and affordably identify the causal mutations from a limited number of mutant lines, providing informative targets for genome editing [7].

This Special Issue has a collection of eight research articles, ranging from crop improvement through induced mutagenesis to creating mutations of multi-copy genes through CRISPR/Cas9-mediated gene knockout. Khan et al. analyzed 206 mutant pools for seed protein content and amino acid composition using wet chemistry and demonstrated that the mutant library can be an important resource to improve seed protein content and essential amino acids [11]. Wang et al. screened the entire USDA sesame collection for high oleic acid lines, which causes sesame to be more healthy and remain stable during storage [12]. Wang et al. identified a single sesame plant from a mixed accession which has 54% oleic acid content compared to the average of 39.5%. Using ethyl methane sulfonate (EMS)-induced mutagenesis, the oleic acid level was further increased to 70% [12]. This line will serve as a critical resource for breeding sesame varieties with high oil quality. Using next-generation sequencing, Zhou et al. conducted a genome survey of *Ilex chinensis*, an ornamental and medicinal plant that has little genomic information available [13]. They discovered over 300,000 microsatellite markers, providing a rich resource for genetic characterization of this valuable plant species. Physical mutagens, such as gamma and X-rays, have been important mutagens for mutation breeding for over 80 years; however, the spectrum of mutations induced by these mutagens is not well understood. Jankowicz-Cieslak et al. revealed the spectrum of mutations induced by gamma- and X-ray in rice using next-generation sequencing [14]. Hudson demonstrated that induced mutation by N-methyl nitrosourea (NMU) is an effective approach to improve protein and oil content in soybean [15]. Karlson et al. described an approach to develop nutritious fresh leaf greens from a tetraploid *Brassica juncea*, or brown mustard [16]. Leaf pungency due to a reaction of the myrosinase enzyme with its glucosinolate substrates is a major factor preventing the use of mustard leaves as nutritious leafy greens. By knocking out the type-I myrosinase multigene family in a tetraploid *Brassica juncea* with CRISPR-Cas12a, Karlson et al. developed a leafy green mustard plant that has a stable reduction in leaf pungency in human sensory and biochemical analyses [16]. Despite the power of isolating mutants of essential genes through conventional mutagenesis, the process can be challenging. Pathak et al. described an approach to create mutants in rice by targeting essential genes for TOR and SnRK1 [17]. Lastly, Neelakandan et al. presented an application for the use of the CRISPR/Cas9 genome-editing system in editing genes in peanut [18].

This Special Issue also includes two excellent review articles. Tillett et al. reviewed the genes affecting grain weight and grain number in wheat, which are two important and often compensatory components of yield [19]. It may be possible to edit those genes using advanced genome-editing techniques to achieve a balanced alternation of grain size and number and achieve optimized grain yield. Niazian et al. reviewed the application of CRISPR/Csas9 root transformation for functional analysis of root traits in soybean [20].

With the rapid development of next-generation sequencing techniques and genome-editing tools, conventional mutagenesis has entered a new phase beyond the release of new cultivars. A high-quality mutant library can be a rich resource to generate and preserve a diverse range of mutations with a limited number of lines. The mutant library can be characterized by both reverse and forward genetics approaches with next-generation sequencing techniques to produce new desirable traits for breeding and causal gene mutations for genome editing. If the causal mutations underlying superior agronomic traits are known, they can be quickly reproduced in elite lines through precise genome-editing techniques with few or no unrelated background mutations, enabling the traits from the mutant library to become quickly applicable to breeding. A true revolution of plant breeding, as well as animal breeding, will emerge with the combination of efficient mutagenesis, high-throughput next-generation sequencing, and precise genome-editing techniques.
